# Emotions, Emotion Management and Emotional Intelligence in the Workplace: Healthcare Professionals' Experience in Emotionally-Charged Situations

**DOI:** 10.3389/fsoc.2021.640384

**Published:** 2021-04-06

**Authors:** Lara Carminati

**Affiliations:** ^1^Faculty of Behavioural, Management and Social Sciences, University of Twente, Enschede, Netherlands; ^2^Surrey Business School, University of Surrey, Guildford, United Kingdom

**Keywords:** emotions, emotional intelligence, emotion management, healthcare professionals, well-being

## Abstract

This perspective article is grounded in a cognitive and context-dependent view on emotions. By considering emotions as socially embedded and constructed, the different but related concepts of Emotion Management and Emotional Intelligence can be introduced. Yet, research juxtaposing and applying them within the healthcare sector to explain healthcare professionals' multifaceted emotional experiences at work is still scarce. Hence, this article contributes to the literature on emotions by offering an overarching perspective on how the juxtaposition of Emotion Management and Emotional Intelligence may help healthcare professionals to bridge the developmental transition between these two crucial abilities which, in turn, can help them overcome emotional difficulties in complex situations. Such integration would positively influence individuals' behavioral and mental health, as well as the overall quality of the healthcare system.

*I don't want to be at the mercy of my emotions*.

I want to use them, to enjoy them, and to dominate them

— *Oscar Wilde, The Picture of Dorian Gray*.

## Introduction

Over the last decades there has been an increased interest on the role of emotions in the multifaceted experience of work (Kluemper et al., 2013). Affective factors (e.g., emotions, feelings, mood etc.) have been recognized not only as pervasive in regulating and guiding human behavior (Von Scheve, 2012), but also directly related to individual and organizational well-being, performance and job satisfaction (Seo et al., 2010). This new tendency seems to clash with the quantification and monetarization principles promoted by the impact of neoliberalism within the public sector organizations (Connell et al., 2009) which, in turn, have devaluated many intangible values of human, social and organizational capital, such as equality, integrity, and attention to the quality of individual interactions (Diefenbach, 2009). And yet, perhaps as a natural reaction to this de-emphasis of emotions in modern society (Scheff, 2014) or to the negative psycho-sociological effects on the people delivering public services, ranging from stress and demoralization to turnover and demotivation (Schrecker, 2016), new attention to emotions has emerged.

This new attention moves away from a dated idea that depicted them as being physiological and primitive perturbations, to embrace a more cognitive and socially embedded view (Fenton-O'Creevy et al., 2011). By doing so, the pivotal notion of Emotion Management (EM), described as an alteration of individuals' emotional *status* to meet those criteria that are deemed most appropriate by the collectivity, can be introduced in the discourse on emotions (Langlotz and Locher, 2013).

EM is often studied in relation to Emotional Intelligence (EI), which is broadly defined as the ability to perceive, facilitate, understand and regulate one's and other's emotions. Despite the increasing importance of these two concepts, research has often neglected to examine how their juxtaposition can have a beneficial impact on individuals' behavioral and mental health in critical circumstances. Hence, through the exemplary, practice-based cases of healthcare professionals facing emotionally-charged situations, the present perspective article contributes to the literature on emotions by offering an overarching view of the importance of integrating EM and EI to positively affect individuals' experiences at work. More specifically, it is argued that the integration of EM and EI is a paramount condition that can bridge the developmental transition between these two crucial abilities, thus helping employees to confront, sustain and overcome extreme circumstances in many employment sectors, but especially in the sensitive and complex field of healthcare.

The article is divided into four main parts. After outlining a short *excursus* on the role of emotions, the key concepts of EM and EI are presented. Examples involving healthcare professionals are then introduced to elucidate the crucial importance of effectively developing EM and EI to tackle exceptionally difficult situations.

## Emotions

Both inner and outer human life produce emotions (Scheff, 2014). Emotions pervade human social affairs and can significantly determine people's experience in their workplaces, influencing their well-being, motivation, job satisfaction and performance (Seo et al., 2010). However, in a sort of Cartesian duality, emotions have been opposed to reason (Lindgren and Packendorff, 2014) due to their irrational, bodily nature^1^ (Fenton-O'Creevy et al., 2011). Considered as instinctive, “internal states of physiological arousal or visceral experiences” (Rosemberg, 1990, p. 3), emotions have been for long relegated to a subordinate position to cognitions and neglected by organizational research. Modernization and Western societies have indeed focused their attention to individuals' rationality and emphasized the cognitive dimension of thoughts and behaviors over the relational, socio-emotional counterpart (Scheff, 2014).

Nonetheless, emotions have been recently seen as products of both organismic and reflexive processes (Fenton-O'Creevy et al., 2011). Functioning as a rationalization process, this reflexive agency interlaces emotions with cognitions, allowing the former to be “felt” *and* “thought” to offer a truthful representation of the experienced world (Rosemberg, 1990; Langlotz and Locher, 2013). Through such cognitive awareness of the self and the contextual others (Israelashvili et al., 2019), emotions become “feelings,” which underline how emotions are socially constructed by means of social interactions, permeated by social influences and embedded in social situations (Langlotz and Locher, 2013).

## Emotion Management (EM)

Viewing emotions as socially constructed implies that individuals may consciously alter their emotional expressions to align them with the expectations of the surrounding context (Beal et al., 2013). This regulating effort is generally called EM but, if carried out within the work domain, then it can be associated to emotional labor^2^ (Hochschild, 1979; Kluemper et al., 2013). Expanded on Goffman's idea that emotions are the results of impression management and thus of individuals' effort and energy to perform in a social situation to avoid embarrassment (Strauss, 1997), Hochschild defined emotional labor as “the act of evoking or shaping, as well as suppressing, feeling in on self” (Hochschild, 1979, p. 561). Emotional labor represents the expression in the workplace of those emotions deemed acceptable by the community to conform to organization expectations (Grandey, 2000; Thwaites, 2017). Indeed, in the work setting employees may be required and even forced to modify their emotional expressions as part of their professional role to enhance organization task, performance and efficiency (Joseph and Newman, 2010; van Dijk et al., 2017).

However, the emotion regulation processes may generate an uncomfortable emotional dissonance indicating a discrepancy between what employees feel and what they ought to feel (Gross, 1998; Thwaites, 2017). Through emotional labor, individuals put a certain degree of effort into adjusting their emotions to the expected ones by inhibiting inner feeling to level off the dissonance (Hochschild, 1979; Von Scheve, 2012). Such effort can be accomplished by two mechanisms: deep acting, a sincere and authentic attempt to shape inner feelings; and surface acting, in which a modification of the displays takes place without altering the inner feelings (Hochschild, 1979; Grandey, 2000).

## Outcomes of Emotion Regulation Processes

These emotion regulation processes may lead to both positive and negative outcomes (Gross, 1998). Whilst at the organizational level these processes are likely to positively improve organizational productivity, performance and the quality of social interactions between parties (Fenton-O'Creevy et al., 2011), at the individual level they are more likely to lead to negative outcomes, given the lack of individuals' spontaneous manifestation of emotions (Von Scheve, 2012). Especially when engaging in surface acting, people dedicate their undivided cognitive attention to it (Grandey, 2000). By drawing from an already limited pool of resources, this conscious process can significantly hamper individuals' performance and be detrimental to their well-being (van Dijk et al., 2017), causing exhaustion and frustration, ego-depletion and stress, fatigue and burnout, as well as reducing self-identity in favor of a pseudo identity (Moon and Hur, 2011; Beal et al., 2013; Zaehringer et al., 2020).

Nonetheless, research has also shown that positive individual outcomes are also possible (Gross, 1998; Hayward and Tuckey, 2011). Indeed, through a gradual and constant learning experience in the workplace (Ybarra et al., 2014), employees may engage in, and develop, automatic responses with minimal participation of cognitive functioning and strain (Fenton-O'Creevy et al., 2011; Szczygiel and Mikolajczak, 2018). In this way, they can “work toward managing naturally emerging emotions to facilitate their emotional, cognitive and physical functioning” (Hayward and Tuckey, 2011, p. 1510). Research, for instance, has shown that automatic regulatory processes may help individuals to engage in EM effortlessly, minimizing the costs and energy involved to fulfill the expectations of their social role (Ybarra et al., 2014; Zaehringer et al., 2020). This ability to automatically understand and efficiently manage one's and others' emotions is called EI (Mayer et al., 2016).

## Emotional Intelligence (EI)

A plethora of sometimes contrasting definitions exists to capture the essence of EI (Ybarra et al., 2014). Sometimes regarded as an ability, other times as a personality trait, or also as a combination of both (Mayer et al., 2016; MacCann et al., 2020), there is general agreement in stating that EI comprises four main hierarchically-arranged components: perceiving emotions in oneself and the others, using emotions to facilitate thoughts, understanding emotions and regulating emotions (Joseph and Newman, 2010; MacCann et al., 2020). Several studies have indicated that EI is positively correlated with individual well-being, job performance, productivity, personal integrity and interpersonal sensitivity (Abe, 2011; Mayer et al., 2016). Thus, EI may play a key role in employees' emotional experience of work, not only because it may facilitate effective workplace functioning and positive outcomes, but also because it may help individuals to enhance their self-identity by fulfilling the expectations intrinsically associated with it (Hayward and Tuckey, 2011).

## EM and EI Into Healthcare Practices

Among the numerous and diversified situations and contexts in which the role of emotions, EM and EI have been explored, the healthcare service is certainly of a particular interest due to its very sensitive and delicate environment as well as the institutional and societal pressures around it (Bailey et al., 2011b). Specifically, the relationship between healthcare professionals and their patients provides a unique situation in which emotion regulation processes can be studied. Indeed, due to the lack of support in coping with stress, the low levels of reward and the frequent emotional interactions with the patients and their family, healthcare professionals constantly need to perform EM (Hayward and Tuckey, 2011; Martin et al., 2015; Szczygiel and Mikolajczak, 2018). Hence, emotion regulation processes are necessary to perform optimally and deliver high-standard quality of care (Kooker et al., 2007; Bailey et al., 2011b; Martin et al., 2015).

One of the primary duties of doctors and nurses is to establish a good relationship with their patients to develop trust and deliver the best possible care (Bailey et al., 2011a). However, especially in emotionally-charged situations, the relationship with patients is extremely delicate. Emotions such as anxiety, anger, helplessness and frustration need to be appropriately managed since the effort of altering emotional *status* would allow healthcare professionals to sustain an outward appearance in line with what they think is the most appropriate behavior to show (Martin et al., 2015; Szczygiel and Mikolajczak, 2018). If ill managed, not only the emotion regulation process may become exhausting (Zaehringer et al., 2020), but it may also stimulate distancing behavior as primary coping mechanism to avoid personal grief (Bailey et al., 2011b). This might explain why doctors and nurses may struggle to demonstrate empathy or a warm, genuine concern (Kooker et al., 2007). For example, Bailey et al. (2011a) noticed that nurses working in an emergency department tended to avoid building a relationship with the patient to protect themselves from potential upsets and deal with loss more easily. By depersonalizing the situation through a process of objectification –“we have some *thing* on the table”– that was removing the personhood of the patient, they could shield and safeguard themselves (Bailey et al., 2011a, p. 366). The authors thus concluded that emergency staff and practitioners, whose role is vital in such delicate moments, are still often unable to perform EM and need support from policymakers and institutions to be able to deliver excellent care in emotionally-charged situations.

Nonetheless, if EM is handled appropriately, healthcare professionals may be able to regulate their boundaries of distance and intimacy in a conscious and more effective way (Bailey et al., 2011b). Doctors and nurses' manipulation of their emotional boundaries can function as a filter through which interact with patients, so that they are physically and cognitively present, whilst controlling their emotional connections (Hayward and Tuckey, 2011, p. 1513). Consequently, prior to initiating an interaction, healthcare professionals would create distance to achieve emotional neutrality as a form of control as well as to evoke a sense of calm and acceptance in others (Israelashvili et al., 2019). From this neutral starting point, they could then strategically determine whether to keep the distance or to connect with patients. An emotional connection would allow doctors and nurses to refuel their energy reservoirs, enhance their personal and professional identity, as well as to experience closeness, empathy and compassion toward patients (Martin et al., 2015). Overall, this would boost excellence in practice (Bailey et al., 2011b).

Once these processes of emotion regulations are internalized, they can become automatic mechanisms that allow healthcare professionals to efficiently manage emotions in critical circumstances. When this is achieved, EI has been developed (Szczygiel and Mikolajczak, 2018). Since EI can generate and increase trustworthiness between healthcare professionals and patients (Kluemper et al., 2013), it represents a constituent element of the emotional fabric of clinical and medical practice. However, developing and appropriately managing EI does require time, patience and experience (Ybarra et al., 2014). Hence, the juxtaposition of EM and EI would allow to bridge the developmental gap between EM and EI, by smoothing the transition between these two crucial abilities. Thus, their integration would remarkably help healthcare professionals to solve problems, facilitate learning, take on risks and cope effectively with environmental demands, especially in emotionally-charged situations (Moon and Hur, 2011).

## Conclusion

Through the exemplary, practice-based case of healthcare professionals facing emotionally-charged situations, this perspective article offered an overarching view of the importance of integrating EM and EI to reinforce each other and promote a beneficial influence on individuals' work experience, even within the restrictions imposed by the quantification and monetarization principles of neoliberalism. More specifically, this paper aimed to show how juxtaposing EM and EI could bridge the gap between these two pivotal abilities and foster individuals' behavioral and mental health. This perspective article therefore contributed to the literature on emotions by arguing that the integration of EM and EI is a paramount condition to help employees to face, sustain and overcome extremely emotionally-charged situations in their workplaces, especially in the sensitive and complex field of healthcare.

## Author Contributions

The author confirms being the sole contributor of this work and has approved it for publication.

## Conflict of Interest

The author declares that the research was conducted in the absence of any commercial or financial relationships that could be construed as a potential conflict of interest.

## Footnotes

^1^From an etymological angle, the word “emotion” stems from the Latin word “*haemo*” (and the Greek word “*haima*”) that means blood, together with the verb “*moveo*” which means “to move,” “to act”: hence, ‘the action of the blood'.

^2^In this perspective article the terms are used interchangeably, with still a preference to use EM as dominant term.
